# The effect of geriatric intervention in frail elderly patients receiving chemotherapy for colorectal cancer: a randomized trial (GERICO)

**DOI:** 10.1186/s12885-017-3445-8

**Published:** 2017-06-28

**Authors:** C. M. Lund, K. K. Vistisen, C. Dehlendorff, F. Rønholt, J. S. Johansen, D. L. Nielsen

**Affiliations:** 1 Department of Medicine, O106 Herlev and Gentofte Hospital, Copenhagen University Hospital, Herlev Ringvej 75, -2730 Herlev, DK Denmark; 2 Department of Oncology, Herlev and Gentofte Hospital, Copenhagen University Hospital, Herlev, Denmark; 3 Danish Cancer Society Research Center, Danish Cancer Society, Copenhagen, Denmark; 4 Department of Clinical Medicine, Faculty of Health and Medical Sciences, Copenhagen University, Copenhagen, Denmark

**Keywords:** Chemotherapy, Colorectal cancer, Comprehensive geriatric assessment, Elderly, Frail, Intervention

## Abstract

**Background:**

Better surgical techniques, chemotherapy and biological therapy have improved survival in patients with colorectal cancer (CRC), most markedly in younger patients. About half of patients over 70 years receive dose reductions or early treatment discontinuation of the planned adjuvant or first-line treatment due to side effects. The Comprehensive Geriatric Assessment (CGA) is a multidisciplinary evaluation of an elderly individual’s health status. This assessment in older patients with cancer can predict survival, chemotherapy toxicity and morbidity.

**Methods:**

This randomized phase II trial (GERICO) is designed to investigate whether comprehensive geriatric assessment and intervention before and during treatment with chemotherapy in frail elderly patients with stages II–IV CRC will increase the number of patients completing chemotherapy. All patients ≥70 years in whom chemotherapy for CRC is planned to start at Herlev and Gentofte Hospital are screened for frailty using the G8 questionnaire at the first visit to the outpatient clinic. The G8 questionnaire is a multi-domain screening tool to identify frail or vulnerable patients at risk of increased toxicity and morbidity. Frail patients are offered inclusion and are then randomized to two groups (the intervention group and the control group). Patients in the intervention group receive a full geriatric assessment of comorbidity, medication, psycho-cognitive function, physical, functional and nutrition status, and interventions are undertaken on identified health issues. Simultaneously, they are treated for their cancer according to international guidelines. Patients in the control group receive the same chemotherapy regimens and standard of care. Primary outcome is number of patients completing scheduled chemotherapy at starting dose. Secondary outcomes are dose reductions, treatment delays, toxicity, time to recurrence, survival, cancer-related mortality and quality of life.

**Discussion:**

This ongoing trial is one of the first to evaluate the effect of geriatric intervention in frail elderly patients with CRC. The trial will provide new and valuable knowledge about whether it is beneficial for the elderly patient undergoing chemotherapy to be treated simultaneously by a geriatrician.

**Trial registration:**

ClinicalTrials.gov ID: NCT02748811. The trial was registered retrospectively; registration date 04/28/2016.

## Background

The incidence of colorectal cancer (CRC) increases with age [1], and as populations are getting older [2], an increasing number of elderly patients will be diagnosed with CRC. In recent years, mortality has decreased in patients with CRC due to better surgical techniques, chemotherapy and new biological therapy, but in elderly patients, the mortality remains higher than in younger patients [3, 4].

Adjuvant chemotherapy after surgery for stages II and III colon cancer (CC) with 5-fluorouracil (5-FU) [5, 6] or capecitabine [7, 8] reduces the recurrence rate and improves overall survival (OS). The evidence for the recommendation of adjuvant chemotherapy in rectal cancer is sparse, but one meta-analysis supports the use of 5-FU-based postoperative treatment [9]. For patients older than 70 years, adjuvant treatment also prolongs time to recurrence [10], and OS is higher in CRC patients >75 years receiving adjuvant chemotherapy compared to patients not treated with chemotherapy [11–13]. There are divergent results in elderly (defined as age > 70 years) regarding the addition of oxaliplatin to 5-FU (FOLFOX) or capecitabine treatment (CAPEOX) on disease-free survival (DFS) and OS [14–16]. However, combination chemotherapy is considered standard therapy for patients with stage III CRC, but the beneficial effect for elderly patients and patients with stage II CC remains controversial [5, 17].

Although the benefit of a least single-agent treatment seems to be the same for elderly and younger patients in clinical trials, elderly patients are less frequently treated [18]. In a study reported by Sanoff HK, 63% of patients aged 75–79 years with CC stage III received adjuvant chemotherapy, whereas only 14% of patients ≥85 years received chemotherapy [11]. Elderly patients do not receive adjuvant treatment because of high age, co-morbidity and poor performance status (PS) [12], but this could also be due to lack of social support and concerns regarding toxicity and efficiency. The underuse of this potentially lifesaving treatment may explain a higher prevalence of recurrence and the higher mortality seen in the elderly with CRC [4]. About half of patients over 70 years receive dose reductions or early treatment discontinuation of the planned treatment due to severe side effects [12].

For patients with metastatic CRC (mCRC), treated with combinations of chemotherapy drugs (5-FU, capecitabine, oxaliplatin or irinotecan) and new biological drugs in clinical trials, the median OS has increased from 6 to 8 months to about 24 months over the last decades [4, 19]. But, in clinical practice, the median survival is only 10–11 months, mainly due to poor OS in elderly patients and patients with low PS who do not receive any treatment [20].

In elderly patients, irinotecan as second-line therapy has the same efficacy as that seen in younger patients, but gives an increased risk of grades 3–4 neutropenia and diarrhea. [21]. Irinotecan/5-FU combinations are well tolerated and their efficacy is similar to that seen in younger patients [22].

Retrospective studies suggest that combination chemotherapy for elderly patients with mCRC is associated with longer progression-free survival (PFS) [23], even if 71% of all patients with CAPEOX received dose reductions. In another study of elderly and frail patients, no significant improvement was seen when reduced doses of oxaliplatin were added to capecitabine or 5-FU [24].

In elderly patients with mCRC, adding bevacizumab to standard chemotherapy improves PFS and OS [25, 26]. However, an increase in thromboembolic events is observed in patients >65 years [25, 27, 28]. For cetuximab and panitumumab, efficacy and safety are similar for patients ≤65 and >65 years [29]. Single-agent (off-label) panitumumab has been shown to be an effective and well-tolerated first-line treatment for frail elderly patients deemed unfit for chemotherapy [30].

### Geriatric oncology

Oncologists and geriatricians have begun to cooperate to individualize and improve treatment for elderly patients with cancer [31]. Elderly vulnerable patients are at high risk of severe toxicity [32]. During the last decade, there has therefore been an increased focus on the importance of individual patient assessment before treatment decisions are made. Chronological age per se should not be an exclusion criterion for adjuvant or palliative chemotherapy [33–35]. Comorbidity and PS more than age alone influence treatment outcomes for both patients in adjuvant [36] and palliative therapy [23, 37]. The Comprehensive Geriatric Assessment (CGA) is a multidisciplinary comprehensive evaluation of an elderly individual’s functional status, physical performance, comorbidity, polypharmacy, cognitive and emotional function, nutritional status and need of social support [38, 39]. CGA in older patients with cancer can predict survival, chemotherapy toxicity and morbidity [40–42]. The assessment can detect unknown geriatric problems and lead to change in treatment strategy for 20% to 49% of patients [43, 44]. To assess all those important factors, there is a need for a multidisciplinary approach in decision making, before and during treatment with chemotherapy, in elderly patients with CRC [31, 35].

Geriatric frailty assessment can predict 1- and 5-year survival in patients after surgery for CRC, and the impact of frailty on 5-year survival is comparable with the impact of TNM stage [45]. Interventions on identified health issues found by the CGA could reduce the side effects and thus increase the number of patients who complete chemotherapy. The supportive care could also improve coping strategies in dealing with adverse events and thereby improve treatment compliance. This could increase survival of the patients. In a recent randomized study, the impact of geriatric intervention on tolerance to chemotherapy in elderly patients (age > 70 years) was investigated. The patients who received CGA in the intervention group (*N* = 46) were more likely to complete planned chemotherapy without dose modifications, but there was no difference in grade 3+ toxicity between the two groups [46].

### Screening for frailty

CGA is considered as the most appropriate way to examine the overall health situation of the elderly patient, but it is time consuming and not required for all patients, and other frailty screenings tools have been developed and validated. In the recommendations from the International Society for Geriatric Oncology (SIOG) from June 2014 [47], the G8 questionnaire is found to be the more or equally sensitive than other screening tools compared to CGA. For patients with an abnormal G8 score (≤ 14/17), full CGA is recommended [48].

### Geriatric intervention in non-oncological settings

The effect of geriatric intervention in patients undergoing chemotherapy for CRC has not been fully investigated. However, there is evidence for a beneficial effect of geriatric assessment for elderly admitted to hospitals for medical conditions. In a meta-analysis of randomized trials, CGA after appropriate interventions during hospitalization increased the likelihood of being alive and living at home 6 months after hospital discharge [49]. The beneficial effect was greatest for geriatric wards, but geriatric teams had also a positive effect.

Several studies have evaluated the cooperation between geriatricians and surgeons from different specialties, and a beneficial effect in terms of cost reduction and treatment outcomes was found, e.g. in elderly patients with hip fractures randomized to comprehensive geriatric care compared to usual orthopedic care, 4-month mobility improves [50]. Multi-disciplinary preoperative CGA with post-operative in-ward follow-up by geriatricians in elderly patients at risk of complications due to elective orthopedic surgery decreased the risk of complications and reduced length of hospital stay [51]. For elderly patients undergoing elective orthopedic, urological or gastrointestinal surgery, a preoperative CGA led to fewer cancelled procedures, a decrease in postoperative complications and a reduced length of stay [52].

To our knowledge, there are no intervention studies evaluating the beneficial effect of geriatric intervention on chemotherapy completion in patients undergoing chemotherapy for CRC. No such studies are described on clinical trials.gov (search July 15, 2016). The present trial will therefore provide new and valuable knowledge about whether it is beneficial for the elderly patient with CRC to be simultaneously treated by a geriatrician.

There is no evidence for the effect of the full geriatric intervention in patients with CRC undergoing chemotherapy; however, different parts of the geriatric assessment have been shown to be predictive, and intervention has been shown to be beneficial.

### Physical exercise

In the last decade, there has been an increased focus on physical performance, exercise and their relation with a diagnosis of CC, treatment outcomes and quality of life during and after chemotherapy. Physical activity prevents risk of CC [53], and several studies suggest that physical activity after cancer diagnosis increases DFS and OS [54], and it is found to be associated with CC-and CRC-specific mortality [55, 56]. In mouse models, physical exercise reduces tumor growth, probably by increasing immune cell infiltration [57]. Jung et al. found that decreased muscle mass at the start of treatment was significantly associated with toxicity grades 3 to 4 in patients undergoing adjuvant chemotherapy [58]. Others have reported the beneficial effect of physical training on quality of life and fatigue during chemotherapy [59, 60]. In a recent study of 33 patients with CC, an 18-week exercise program significantly reduced physical and general fatigue [61]. In patients undergoing adjuvant chemotherapy for CRC, supervised exercise compared to usual care was superior in improvements of physical activity level, functional status and QOL, but the patients were younger than in GERICO (mean 56.5 years) [62]. Among breast cancer patients undergoing adjuvant treatment, both moderate to high-intensity exercise and low intensity home training programs resulted in better physical performance but also less pain and toxicity (e.g. nausea and vomiting) due to the treatment [63]. In patients med lung cancer, home-based walking has been shown to be feasible and have a beneficial effect on anxiety and depression [64]. In a meta-analysis, Meneses-Echáves et al. conclude that supervised physical activity interventions reduce cancer-related fatigue and suggest that combined aerobic and resistance exercise regimens should be included as a part of rehabilitation in people diagnosed with cancer [65].

Many elderly patients suffer from sarcopenia, an age-related decrease in muscle mass and strength leading to decline in physical performance. Sarcopenia is associated with falls, disabilities and increased risk of death [66]. Muscle strength is measured with handgrip strength, with a cut off of 20 kg for women and 30 kg for men [67]. Muscle mass can be measured with DXA scanning, and physical performance is assessed by different tests. Usual gait speed, which is commonly used, is associated with OS [68], and a gait speed over 4 m (at a threshold of 1 m/s) can predict adverse outcomes in community-dwelling older people [69]. Gait speed is also recommended as a frailty screening test in cancer patients 65 years of age and older [70]. Low handgrip strength and slow usual gait speed are independently associated with higher mortality [71].

### Nutritional intervention

Loss of weight in patients with cancer is associated with a poor prognosis [72], and nutritional support to malnourished patients with gastrointestinal cancer is recommended [73]. There are few studies evaluating the effect of nutrition intervention in cancer patients. In 358 patients with metastatic or locally advanced gastrointestinal, non-small cell lung cancer or mesothelioma undergoing chemotherapy, nutritional advice and supplements had no impact on 1-year mortality or quality of life [74]. In a meta-analysis of 1414 malnourished patients with different cancers included in 13 randomized controlled trials, nutritional intervention was found to be effective in increasing nutritional intake and improving some aspects of QOL, but the interventions had no effect on mortality [75]. In a randomized trial of 336 patients with cancer at risk of malnutrition, dietary counseling increased nutritional intake but had no impact on mortality, toxicity or other treatment outcomes [76]. The authors suggest that cancer cachexia anti-anabolism is responsible for the lack of effect.

## Methods /Design

### Aim

The aim of the study is to investigate whether frail elderly patients (defined as age ≥ 70 years) with stages II–IV CRC will benefit from full comprehensive geriatric assessment and intervention before and during treatment with adjuvant or palliative first-line chemotherapy. We will evaluate whether optimizing all health conditions can increase the number of patients completing 6 months of adjuvant chemotherapy or first-line chemotherapy until disease progression.

### Hypothesis

Our hypothesis is that geriatric intervention can optimize the health and functional status of frail elderly patients with stages II–IV CRC. Due to this geriatric intervention, more patients will receive the scheduled doses and series of chemotherapy. As a consequence, the patients will have a longer OS and an improved quality of life.

### Design

The study is an open, randomized, prospective trial performed at the departments of oncology and medicine at Copenhagen University Hospital, Herlev and Gentofte, Denmark.

### Participants

A total of 140 participants will be included: 70 participants in the intervention group and 70 participants in the control group.

All patients ≥70 years [77] who meet the criteria to either receive adjuvant chemotherapy after surgery for stage III and high-risk stage II CRC or palliative first-line chemotherapy for inoperable or metastatic CRC will be screened for frailty with the G8 questionnaire [78] at their first visit to the oncology outpatient clinic. Frail patients, defined as patients with ≤14/17 points in the G8 questionnaire, will be offered inclusion in the study. Other inclusion criteria are PS 0–2 and a life expectancy ≥3 months. All patients must provide informed signed consent to be eligible for inclusion. Exclusion criteria are other malignancies during the last 5 years (except basal cell carcinoma, squamous cell carcinoma in situ and cervical cancer), and simultaneous participation in a trial of a medical product. The patients with recurrence or metastatic disease must not have received prior adjuvant treatment in the GERICO protocol.

To achieve adequate participant enrolment, the single center research team notifies the oncologists about all patients eligible for screening. The research team obtains informed consent and collects all data.

### Randomization

After inclusion, the participants are randomized to either the intervention group or the control group. The randomization is made using ARRACT (A Real-time Randomization Application for Clinical Trials), a computer program developed and operated by the Department of Oncology Clinical Research at Herlev and Gentofte Hospital. The method used is "Stratified Balanced Allocation Method (n- Treatments)" [79, 80]. In the randomization, the PS of the patients (0 or 1+), and chemotherapy treatment (adjuvant or metastatic setting) are used as stratification variables.

### Description of the two groups

The patients in the control group receive standard treatment with 6 months of adjuvant chemotherapy after surgery for CRC or palliative first-line chemotherapy until disease progression, operation or other scheduled change in treatment. If the patient has other health problems, those issues will be assessed either by the oncologist or by the general practitioner according to standard of care.

The patients in the intervention group also receive standard treatment with 6 months of adjuvant chemotherapy after surgery for CRC or first-line chemotherapy until disease progression, operation or other scheduled change of treatment. These patients will simultaneously be examined and treated by a geriatrician (CL), who will assess all other health issues.

Patients in both groups will fill out quality of life questionnaires (QLQ) prior to the start of chemotherapy, after 2 months, and at the end of the treatment using the validated QLQ C30 and QLQ ELD14 questionnaires from the European Organization for Research and Treatment of Cancer (EORTC). The adverse events are routinely recorded by the oncologist using the standardized CTC criteria version 4.0.

### Geriatric assessment and intervention

All patients in the intervention group will receive full geriatric assessment and a clinical examination at start of chemotherapy, after 2 months, and more frequently if needed. Invention will be performed on identified issues (Table 1).

**Table 1:** The geriatric assessment performed at the start of treatment

Domain	Assessment	Possible intervention
Multimorbidity	Medical record review Clinical examination	Treatment or referrals
Medication	Assessment of medication list based on START/STOPP criteria	Discontinuation Change in dosage Change in prescription of medication
Psychological function	Geriatric depression scale (GDS)	Referral to therapy or medication
Cognitive function	Minimal Mental State Examination (MMSE)	Cognitive evaluation Medication Social support
Nutritional status	Local nutritional screening based on minimal nutrition assessment (MNA)	Referral to dietitian
Physical function	Gait speed 10 m: (cut off 1 m/s) Handgrip assessed with the Jamar Dynamometer: (cut off 20 kg for women and 30 kg for men)	Referral to physiotherapist and scheduled program
Functional status	Activities of daily living (ADL) Instrumental activities of daily living (IADL)	Social support Occupational therapy assessment for equipment needs Transport support
Laboratory parameters	TSH, cbalamin, folat, albumin, vitamin D	Treat deficiencies

In the present study, all patients in the intervention group are screened, as part of the geriatric assessment, with a Jamar Dynamometer for handgrip strength (cut off 20 kg for women and 30 kg for men) and with a 10-m gait speed (cut-off 1 m/s). With a test result under cut off, patients are referred to a standardized physical exercise program twice weekly at the hospital and home exercise once weekly for 12 weeks.

Furthermore, the patients in the intervention group are screened for risk of malnutrition, and the patients are referred to a dietitian if needed.

Review of patients’ medication lists will be performed with focus on polypharmacy and interactions [81] and inappropriate medication based on START STOPP criteria [82].

### The oncological treatment

The standard adjuvant chemotherapy is outlined in Table 2. The standard dose is based on body surface area (BSA), with a possible 25% primary dose reduction in frail patients.

**Table 2:** Regimens and doses of chemotherapy

Adjuvant setting			
Regimen	Drug	Dose	Frequency
Capecitabine	Capecitabine	2000 mg/m^2^ p.o. daily for 14 days	Every 3 weeks*
5-FU	5-FU Calcium folinate	400 mg/m^2^ i.v. Infusion 46 h: 2400 mg/ m^2^ i.v. 400 mg/m^2^ i.v.	Every 2 weeks**
Capeox	Capecitabine Oxaliplatin	2000 mg/m^2^ p.o. daily for 14 days 130 mg/m^2^ i.v.	Every 3 weeks*
Folfox	5-FU Calcium folinate Oxaliplatin	400 mg/m^2^ i.v. Infusion 46 h: 2400 mg/ m^2^ i.v. 400 mg/m^2^ i.v. 85 mg/m^2^ i.v.	Every 2 weeks**
Metastatic setting			
Regimens	Drug	Dose	Frequency
Capecitabine	Capecitabine	2000 mg/m^2^ p.o. daily for 14 days	Every 3 weeks*
5-FU	5-FU Calcium folinate	400 mg/m^2^ i.v. Infusion 46 h: 2400 mg/ m^2^ i.v. 400 mg/m^2^ i.v.	Every 2 weeks**
Capeox	Capecitabine Oxaliplatin	2000 mg/m^2^ p.o. daily for 14 days 130 mg/m^2^ i.v.	Every 3 weeks*
Folfox	5-FU Calcium folinate Oxaliplatin	400 mg/m^2^ i.v. Infusion 46 h: 2400 mg/ m^2^ i.v. 400 mg/m^2^ i.v. 85 mg/m^2^ i.v.	Every 2 weeks***
Irinotecan	Irinotecan	200 mg/m^2^ i.v.	Every 2 weeks****
Capiri	Capecitabine Irinotecan	1600 mg/m^2^ p.o. daily for 14 days 200 mg/m^2^ i.v.	Every 3 weeks *
Folfiri	5-FU Calcium folinate Irinotecan	400 mg/m^2^ i.v. Infusion 46 h: 2400 mg/ m^2^ i.v. 400 mg/m^2^ i.v. 180 mg/m^2^ i.v.	Every 2 weeks****
Irox	Irinotecan Oxaliplatin	165 mg/m^2^ i.v. 85 mg/m^2^ i.v.	Every 2 weeks****

*maximum 8 series

**maximum 12 series

i.v. intravenous, p.o. per os

*optional addition of bevacizumab 7.5 mg/m^2^ i.v

**optional addition of bevacizumab 5 mg/m^2^ i.v

***optional addition of bevacizumab 5 mg/m^2^ i.v., irinotecan 165 mg/m^2^ i.v., cetuximab 500 mg/m^2^ i.v., or panitumumab 6 mg/kg i.v

****optional addition of bevacizumab 5 mg/m^2^ i.v., cetuximab 500 mg/m^2^ i.v., or panitumumab 6 mg/kg i.v

### Study objectives

#### Primary outcome

The primary endpoint of the study is the proportion of patients completing scheduled chemotherapy with the same dose as at the start of treatment.

#### Secondary outcomes

Secondary endpoints include adverse events (registered for every chemotherapy cycle after CTC criteria version 4.0), dose reductions, treatment delays (which follow the standardized guidelines of the department), DFS, PFS, OS, CRC mortality and quality of life.

#### Statistical power and analyzes

The power calculation is made based on the expected impact of the geriatric intervention. The proportion of patients who complete the planned treatment is assumed to increase from 50%, the percentage given in the literature [12], to 75% after geriatric intervention. With 70 patients included in each group (total 140 evaluable patients) such an increase can be detected at a 5% significance level with a probability (power) of 87%. The proportion in the two groups will be compared using a chi-square test.

Dose intensity (DI), the cumulative given dose compared to planned total dose per week, and relative dose intensity (RDI), defined as 100* DI/ planned dose intensity (PDI) (mg/ week), will be analyzed.

DFS is defined as time from surgery to time of recurrence or death. PFS is defined as time from start of first-line chemotherapy to date of progression or death according to RECIST criteria 1.1. OS is defined as time from surgery or start of first-line treatment to time of death. CRC mortality will be analyzed in all patients who are followed until outcome of interest or end of follow-up using statistical methods for censored time-to-event data.

All statistical analyzes will be performed by a statistician (CD). The statistical software package R (http://www.rproject.org) is used for all analyses. Disease-free survival, PFS and OS will be estimated with the Kaplan–Meier method and compared with a log-rank test. Additional subgroup analyses based on chemotherapy regimen will be performed if feasible. Missing data will be handled with multiple imputations.

## Discussion

In spite of the high prevalence of CRC and the high incidence of CRC-related mortality, elderly patients are under-presented in clinical trials [38]. Nevertheless, elderly patients are the largest group of cancer patients, and their number is rapidly growing.

When treating elderly patients with comorbidity and poor PS, oncologists today possess a limited number of treatment options. Due to concerns regarding toxicity and the lack of guidelines for treating elderly patients, there is a trend toward less aggressive treatment and exclusion of elderly patients especially in the adjuvant setting, where chemotherapy is potentially lifesaving [83]. There is, however, evidence that chemotherapy is also effective in elderly patients, and in order to improve the poorer cancer prognosis, it is of great importance to find the elderly patients who will profit from chemotherapy and to perform dose adjustments in case of side effects [84, 85].

The cooperation between geriatricians and oncologists in order to individualize and improve treatment for elderly patients with cancer could be the solution. However, there is a lack of randomized intervention studies that evaluate the effect of geriatric intervention treatment outcomes in elderly patients with CRC.

In the present GERICO study, only elderly frail patients with CRC receiving chemotherapy are included. There may be a group of patients who are deemed too frail, too old or to have too much comorbidity to receive chemotherapy, which could profit from the geriatric assessment. An optimization of health conditions in those patients could bring them into consideration for treatment.

Our long-term aim is to integrate the geriatric function into the oncology setting. Optimizing the overall health of elderly patients will potentially allow more intensive treatment, which would lead to increased survival and a higher quality of life. The project will provide new and valuable information for better treatment of frail, elderly patients with CRC.

### Trial status

**Fig. 1.**
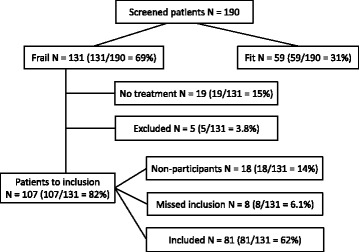
Trial status

The first participant was included in April 2015. The recruitment period is 2–3 years. The last participant included is expected in June 2018. A total of 190 patients have been screened for frailty with the G8 questionnaire, and (131) 69% of the patients were frail, a little lower percentage than seen in other studies [86]. To date, 81 patients have been included. The number of included patients is lower than expected. One of the reasons is that 32 patients were not screened at all, since they were found to be too frail to receive treatment. Of the 131 patients screened frail with the G8 questionnaire, 19 patients did not start any treatment due to performance status, high age, comorbidity or patients’ refusal (Fig. 1). An additional five patients were excluded because of other malignancies. Since the start of the trial, eight patients that could have been included were missed.

## Abbreviations

BSA: Body surface area
CC: Colon cancer
CGA: Comprehensive geriatric assessment
CRC: Colorectal cancer
DFS: Disease-free survival
i.v.: Intraveneus
mCRC: metastatic CRC
OS: Overall survival
p.o.: Per os
PFS: Progression-free survival
PS: Performance status
QLQ: Quality of life questionnaire
